# Nature and Mindfulness to Cope with Work-Related Stress: A Narrative Review

**DOI:** 10.3390/ijerph19105948

**Published:** 2022-05-13

**Authors:** Elisa Menardo, Donatella Di Marco, Sara Ramos, Margherita Brondino, Alicia Arenas, Patricia Costa, Carlos Vaz de Carvalho, Margherita Pasini

**Affiliations:** 1 Department of Human Sciences, University of Verona, 37129 Verona, Italy; margherita.brondino@univr.it (M.B.); margherita.pasini@univr.it (M.P.); 2 Department of Social Psychology, Universidad de Sevilla, 41018 Seville, Spain; ddimarco@us.es (D.D.M.); aarenas@us.es (A.A.); 3 DINÂMIA’CET-IUL, Iscte-Instituto Universitário de Lisboa, 1649-026 Lisboa, Portugal; sara.ramos@iscte-iul.pt; 4 Business Research Unit (BRU-IUL), Iscte-Instituto Universitário de Lisboa, 1649-026 Lisboa, Portugal; patricia_costa@iscte-iul.pt; 5 GILT—School of Engineering of the Porto Polytechnic, 4200-072 Porto, Portugal; carlos_carvalho@virtual-campus.eu

**Keywords:** work-related stress, mindfulness, nature, review

## Abstract

In recent years, work-related stress has grown exponentially and the negative impact that this condition has on people’s health is considerable. The effects of work-related stress can be distinguished in those that affect workers (e.g., depression and anxiety) and those that affect the company (e.g., absenteeism and productivity). It is possible to distinguish two types of prevention interventions. Individual interventions aim at promoting coping and individual resilience strategies with the aim of modifying cognitive assessments of the potential stressor, thus reducing its negative impact on health. Mindfulness techniques have been found to be effective stress management tools that are also useful in dealing with stressful events in the workplace. Organizational interventions modify the risk factors connected to the context and content of the work. It was found that a restorative workplace (i.e., with natural elements) reduces stress and fatigue, improving work performance. Furthermore, practicing mindfulness in nature helps to improve the feeling of wellbeing and to relieve stress. In this paper, we review the role of mindfulness-based practices and of contact with nature in coping with stressful situations at work, and we propose a model of coping with work-related stress by using mindfulness in nature-based practices.

## 1. Introduction

Several institutions and researchers have highlighted global stress prevalence and costs [1,2,3,4]. Hassard [5] presented a systematic review of the available evidence examining the cost of work-related stress (WRS) in Australia, Canada, Denmark, France, Sweden, Switzerland, the United Kingdom, and EU-15. The findings reveal that the total estimated cost of WRS was considerable and ranged substantially from USD 221.13 million to USD 187 billion. The cost of work-related stress within the EU-15 for 2014 was estimated to be EUR 26.47 billion [3]. In Australia, the WRS’s costed USD 14.81 billion yearly [6]. The American Institute of Stress estimated that US businesses lose up to USD 300 billion yearly due to WRS [7]. The economic costs of WRS in Chinese or Asian contexts remain unclear. The only available study reported data from Hong Kong and estimates the cost of WRS range from USD 614 million to USD 900 million [8]. Moreover, productivity-related losses contribute to the majority of the total cost of WRS (between 70 to 90%), with healthcare and medical costs constituting the remaining 10 to 30% [5]. Improved productivity and a healthy workforce are good for the companies’ economy and society. Additionally, it has been also emphasized that stress prevention represents several benefits for companies [2,4].

Different stress management interventions are offered to help keep individuals living under heavy stress to avoid developing more severe stress-related disorders [9]. Stress management programs at workplaces typically focus on psychosocial environments and do not address the growing body of research on workspace’s environmental psychology [10,11]. However, studies show that office designs influence employees’ stress-level, productivity, and wellbeing [12,13,14,15,16]. One aspect of the physical environment that is often overlooked in a traditional office design is the presence of nature or biophilic designs—such as indoor plants, views to outdoor nature, easy access to outdoor nature, or visual representations of nature [17]. Biophilic designs have received increased attention in recent years and are being hailed as a workplace strategy for reducing stress while at the same time enhancing performance and overall wellbeing [18,19,20]. Existing theories, such as the biophilia hypothesis [21], the attention restoration theory [22,23], and the stress restoration theory [24,25], suggest that contact with nature can influence both productivity and wellbeing.

Another kind of stress management intervention acts at the individual level by improving the individual’s capacity to deal with stressors (e.g., physical exercise, counseling, and biofeedback). In this paper, we will focus on the practice of mindfulness for two reasons. First, mindfulness training is a multimodal intervention informed by the principles of positive psychology, with a central focus on skills that enable more effective coping and stress reduction. Second, this paper aims to build a solid framework based on theoretical and empirical evidence to plan an innovative solution for the issue of WRS, proposing Mindfulness-in-Nature-based Intervention (MiNBI) that join together the healing effect of nature and the distressful effect of Mindfulness. 

The current narrative review synthesizes mechanisms underlying these two strategies (nature and mindfulness) and explores how researchers have applied these methods to enhance employees’ health and to manage (prevent and reduce) work-related stress. In the discussion, we propose a hypothetical model for coping with WRS by applying MiNBI.

## 2. Definition of Stress and Work-Related Stress

Stress started to be defined from a biological perspective and is focused on the physiological reaction to stressful factors. In the base of this approach, we can find Selye’s definition of stress [26] as a response to aggression due to an internal or external stressor factor in order to resist, adapt, and restore the individual’s internal equilibrium. Currently, it is accepted that stress indicates a state of elevated activation of the autonomic nervous system with affective, cognitive, and behavioral manifestations [27] and that an individual can feel this activation when he perceives their resources and capabilities as inadequate to handle the hassles and difficulties in an environment [28], shining a light on coping mechanisms.

Over the years, the focus shifted from cause–effect linearity to the interactions between the individual and the context (physical and social) [29,30,31]. Seyle’s theory was criticized since it devalues the role of the cognitive dimension involved in the answer [32]. Indeed, individuals can interpret certain events or stimulus as stressful based on their evaluation of the situations, on their past experiences, and on their emotions. The appraisal process involves a continual monitoring of the person’s transactions with their environment in terms of demands, abilities, competence, constraints, and support [33]. In this perspective, stress is a dynamic process in which both individual and environment act one over the other based on a sequential evaluation of the situation. The individuals first evaluate the situation, distinguish what is positive or pleasant from what is aggressive, and interpret this aggression as a challenge, a threat, or a loss. After this, individuals evaluate their own resources to deal with a singular situation. These sequential steps are influenced by emotions, which make the process even more complex and dynamic across time [33]. 

Stress, as a research topic, arrives at the field of psychology by Murrel [34] who defined occupational stress as a pression or external charge that causes tension (or strain). Currently, in the literature, WRS is also termed in the literature as ‘job stress’ or ‘occupational stress’, and generally refers to situations faced in the workplace context that are related to overload, high demands, or expectations [35]. As it happens for stress in general, several definitions of work stress can be found, and most of them retain the main definition of stress as an individual reaction while placing it in the dynamics of work contexts. A consensual definition of work stress is presented in an official report from the European Commission, stating that it refers to the emotional, cognitive, behavioral, and physiological reaction to aversive and noxious aspects of work, work environments, and work organizations. It is a state characterized by high levels of arousal and distress and often by feelings of not being able to cope [32].

Stress has been largely studied in the work psychology domain, exploring the main causes or stress factors that can be present in the working contexts and also emphasizing the consequences for individuals and organizations [36]. 

Regarding stressors, the UK government’s health and safety division proposed a model, the Health and Safety Executive (HSE), that covers six main areas of work design that could be causes of stress: (1) Demands (includes workload, work patterns, and work environment); (2) Control (relates to the degree of decision that people have over the way they work); (3) Support (includes encouragement, sponsorship, and resources provided by the organization, line management, and colleagues); (4) Relationships (includes promoting positive working to avoid conflict and dealing with unacceptable behavior); (5) Role (workers’ understanding their role within the organization and having clear and non-conflicting roles); (6) Change (relates to the way changes are managed and communicated within the organization) [37].

Additionally, we should not forget the stressors that can emerge from the content of work and from the ergonomic design of the workplace, such as the distribution of work, task changes, changes of machines or other devices, noise, dust, temperature, intensity and fatigue, or the use of uncomfortable personal protective equipment [38,39]. 

Considering the main reported consequences, a systematic review of WRS conducted by Burman and Goswami [35] highlights the following: cognitive consequences (mental illness, lack of concentration and judgment capacity), behavioral consequences (sleeping disorders, poor eating habits, addictive consumption of drugs or alcohol, and neglecting responsibilities), emotional consequences (restless, irritation, impatience, anxiety, isolation, and depression), and physical consequences (high blood pressure, nausea, cardiovascular problems, back, and neck pain). In addition to individual consequences, there are also negative consequences for the organizational side: weak commitment, low engagement, unsafe behaviors, low performance, absenteeism, and turnover [36].

## 3. Nature and Work-Related Stress

Physical settings can play a role in coping with stress. A rapidly growing amount of research from many different areas (e.g., environmental/consumer/health/sport/organizational/occupational psychology, environmental epidemiology, and public health) indicates that nature has a multiplicity of beneficial qualities for mental health [40,41], including stress reduction [42,43,44,45]. With the term “Nature”, we refer to spaces (outdoor and indoor) and views that incorporate natural elements (e.g., trees, rivers, and beaches), material (e.g., wooden and water), sound (e.g., birds and water), or odors (e.g., essential oils). 

To recover from stress, individuals need to replenish their lost resources by engaging in activities that either restore old resources or generate new resources [46]. This process can be described as passive recovery, which follows from the relief from work demands [47]. As claimed by the Attention Restoration Theory [23] and Stress-Recovery Theory [25], exposure to nature restores emotional and cognitive resources, permitting stress reduction. Thanks to the natural elements that capture the workers’ involuntary attention and make feel him/her “being-away”, the workers can receive relief from work demands and restore their resources.

Based on the homogeneous, substantial, and statistically significant findings, there is strong evidence concerning emotional and cognitive changes [48,49,50]. Exposure to natural scenes moderates the negative effects of stress, reducing the negative mood state, and simultaneously enhances positive emotions [48] and the recovery of cognitive performance [49] more than urban environments do. Indeed, the evidence concerning perceived stress level measures is weaker, although they are mainly positive [50]. 

The most common research design has investigated the presence of indoor plants. Two reviews concluded that, in general, the presence of indoor plants appears to reduce stress [51,52]. Studies have been conducted by focusing on specific independent variables. For example, researchers examined the size and volume of greenery [53]; the number of plants installed [54,55]; the shape, size, type, and distance of the plants from the participant [56,57]; and the index of the greenness of interior space [58]. Bakker and Voordt [51] further noted that little attention had been paid to the type of plant or its state of health. The majority of the studies have been conducted in laboratory or quasi-office design [59,60,61,62,63]. A more limited number of studies targeting office workers in real office settings have also been conducted [53,64,65,66,67,68,69,70]. In these studies, indoor plants were placed on the floor, windowsills, shelves, desks, or all of these office options to provide visual access to plants. Most previously mentioned studies focused on the psychological and physiological effects of passive interaction with plants. Nishina [53], however, studied the effects of active involvement with plants. He reported that allowing participants to choose and care for the plants in the study enhanced their satisfaction and contributed to the mitigation of stress in the workplace. Considering physiological effects, green plants in an internal space induced parasympathetic activity, the greater stabilization of the autonomic nervous system, and increased electroencephalographic (EEG) activity [71]. However, due to a low number of studies, the evidence of the physiological effects related to stress recovery is more equivocal [50,72].

Moreover, the view (of real nature or aesthetical images) and natural materials (e.g., wood) reduce stress and anger in a working environment and help workers feel happier and healthier [10,62,73,74]. Moreover, individual preferences for a material can lead to stress reduction [75,76]. Participants preferring wood panels to white steel panels showed greater stress reduction when exposed to their preferred material: wood [75].

Finally, it has recently been discovered that the reduction in stress and improvements to the immune system produced by forest visits can be replicated when evergreen oils are removed from trees, aerosolized, and infused in interior spaces [77]. The essential oils emitted by evergreen trees are associated with improvements to the immune system and the production of natural killer cells [78,79]. These effects could be long-term but vary based on gender, with immune functions increased for up to 30 days in men and only seven in women [80]. Women also report higher stress levels than men, and they are less likely to use outdoor natural environments during the work day [20]. Thus, including access to indoor natural environments as become an even a greater priority for female knowledge workers.

In summary, the literature has shown that, independently from the type of exposure (plants, poster, slides, video, virtual reality (VR) settings, or views of natural environments/stimuli), people experience a general reduction in symptoms related to psycho-physiological stress [42,81]. However, for the emotional aspect, the results are less clear. Indeed, a meta-analysis showed a larger effect for exposure to real nature vs. laboratory simulations [48].

Despite many existing studies and reviews, no existing systematic reviews investigate experimental studies on the effect of contact with nature on healthy adults’ workplace-related outcomes. Moreover, earlier research on workplace design primarily focused on the physical arrangement of employees’ immediate work area and the environmental qualities of the work area. Less attention has been given to building organization, exterior amenities, and site-planning [82].

Greater consideration has been directed toward the multifactorial nature of sound perception and the restorative impact of positively evaluated soundscapes on stress recovery and physio-psychological wellbeing—e.g., the sound of trickling water or birds chirping.

Finally, some questions have not been sufficiently answered with respect to the office setting:

- Does biophilic design actually lead to these outcomes?
- Do all nature contact types lead to equal outcomes?
- How much “nature” is enough or recommended to achieve potential effects?

Designing healthy workplaces, for example, by increasing the possibility of being in contact with natural elements, is only one of the strategies for coping with stress that companies and workers could follow. Another form of coping strategy acts at the individual level by training individuals that are more exposed to stressors and developing and improving their capacity to deal with them in order to be protected from their effects. In the following sections, we review mindfulness techniques that have been found to be effective in modifying the cognitive assessment of the potential stressor, thus reducing its negative impact on health.

## 4. Mindfulness and Work-Related Stress

Mindfulness is defined as “a process of openly attending, with awareness, to one’s present moment experience” [83]. Several definitions have been given of mindfulness, but all of them share two characteristics: Firstly, mindfulness focuses on the awareness in individual’s present experience in terms of external and internal present-moment states (e.g., sounds, body sensations, thoughts, and emotional reactions), which is also called “watchfulness” [84]. Secondly, mindfulness involves adopting an open attitude toward one’s experience [83,85]. 

Mindfulness refers to both the state of mind, which can be considered as a dispositional trait according to the frequency people experienced it at [86], and the practices (included into intervention programs) to foster it [87]. Studies on mindfulness at work can be distinguished in two broad groups: those that focus on state or trait mindfulness, according to the frequency in which workers experience mindfulness, and those which focus on mindfulness-based interventions and its effects at individual and organizational levels. The following paragraphs will present the main findings on mindfulness and wellbeing at work.

Research on trait mindfulness has shown its benefits in terms of greater workers’ wellbeing [88,89,90,91] and as protective factor [87]. For instance, Roche and colleagues [90] found that leaders’ mindfulness is negatively related to their levels of anxiety, depression, negative affect, emotional exhaustion, and cynicism. In another study, Schultz and colleagues [91] showed the direct negative relationship between mindfulness and “work ill-being” is measured in terms of burnout, turnover intentions, and absenteeism. Diary studies demonstrated its positive effects on reducing emotional exhaustion and improving sleep quality [89,92]. A recent meta-analysis [88] showed a significant and positive relationship between trait mindfulness and physical, mental health, and emotion regulation and a negative relationship with anxiety, depression, negative emotions, or burnout.

Mindfulness-based interventions also play a beneficial role in reducing work-related stress and enhancing wellbeing [89,93,94]. The high quantity of studies about mindfulness at work led to a greater production of systematic review and meta-analyses [95]. For example, Lomas and colleagues’ meta-analysis [87] showed a strong negative effect size for health, stress, anxiety, and distress; small to medium positive effect sizes were observed for compassion, empathy, mindfulness (state), and positive wellbeing (life satisfaction, positive affect, and resilience). A non-significant effect size was shown for emotional regulation and depression. Virgili [96] found similar results across intervention types, program durations, and occupations, and the effects were maintained five weeks after the intervention. 

Finally, even if the researchers focused on positive outcomes, mindfulness in the workplace could also have some negative consequences for workers, which must be considered [97]. For example, mindfulness increases creativity, which is helpful in some tasks, but, at the same time, it could increase the duration of most repetitive and mundane tasks [86]. Moreover, in five experiments conducted by Hafenbrack and Vohs [98], a single mindfulness practice weakened energy (arousal) and task motivation. The induction of a mindfulness state reduces energy directed toward accomplishing upcoming tasks [85] and, consequently, task motivation [99,100]. Furthermore, in a recent review Bartlett and colleagues [101] found that highly committed mindfulness training (e.g., 10 h training plus 30 minutes’ daily homework) could increase, rather than decrease, employees’ stress, especially if the training was expressly built for “at risk” employees [102,103].

Research has tried to explain the mechanisms that lead mindfulness to the positive outcomes described above. Literature suggests that in the relationship between mindfulness and WRS, one of the key processes of mindfulness is “decoupling the self from experiences, events, and mental processes” [86,104]. When the self (i.e., ego, self-esteem, and self-concept) is deeply rooted in negative thoughts, emotions, and experiences, people perceive adverse events as more threatening and could feel that their values are under attack [104,105]. On the contrary, mindfulness allows people to select and observe those stimuli that trigger emotions, affecting how they appraise and react. By utilizing mindfulness, people learn to notice and observe stimuli (internal and external) without judging them. This mindfulness approach, in turn, allows people to create a distance between the self and the situation that leads them to have negative thoughts, emotions, and experiences as transient [86,106] and, consequently, to perceive the negative situation as less threatening [103]. Indeed, mindful people show less negative effects after a stressful event [107]. This present-moment nonjudgmental awareness influences, on one side, automatic mental processes by reducing automatic responses and rumination [86,106] that, in turn, lead to better cope with stressful events. On the other side, it also promotes an awareness of one’s physiological state [106] that, in turn, can help individuals better interpret and respond to messages from the body [86]. This leads to a more effective coping mechanisms with stress by reducing reactivity to unpleasant states (lower cortisol levels) [106,108] and allows faster recovery toward baseline levels [108].

Research on mindfulness in the workplace has also received criticism due to the lack of definition consistencies and, in some cases, the lack of rigor in the adaptation of standard intervention programs (e.g., MBSR) to the work environment without guaranteeing the respect of the basic standards of such programs [109]. Another criticism is related to the quality of research designs. For instance, Jamieson and Tuckey’s systematic review [109] showed that 22.5% of the intervention studies revised (9 studies) did not employ a control group; moreover, almost half of the studies (18 studies) failed in the manipulation check. Hence, they did not measure trait and state mindfulness before and after the intervention for each condition/group. The dropout rates of mindfulness interventions and participants’ level of satisfaction with them are lacking in many studies, preventing from identifying the reason why participants do not stay in intervention programs and possible areas of improvements [109]. As other areas of research, another criticism is related with the bias entailed by the self-assessment of state and trait mindfulness, which might be overcome by using triangulation and the use of objective measures according to the mindfulness definition considered. In addition, in most cases, participants were white-collar workforces drawn from large organizations. Therefore, currently, it is not known whether the effectiveness of training differs by setting (e.g., small, medium, and large organization) and role type (e.g., blue collar, administrative, and professional) [101].

Finally, in the organizational context, mindfulness is often used as a “for-gain” approach [110] to gain a healthier and more productive “self”. For example, mindfulness is used as a set of practices for achieving determinate outcomes (e.g., reducing stress or enhancing productivity) [111]. This approach contrasts with the original Buddhist mindfulness approach [111], a “no-gain approach [110], to achieve a not self-centered state of existence [112]. Researchers and those who design mindfulness programs for companies often do not know the theoretical roots of Buddhist mindfulness. Organizational mindfulness programs must not be strictly adherent to Buddhist mindfulness. However, participants could benefit from being aware that mindfulness training should not be used as a single and standalone exercise for reducing stress (or enhancing performance). Mindfulness training should be seen as a gradual and personal process that involves many other factors (e.g., physical, emotional, cognitive, spiritual, ethical, and social) and should lead to adopting a different lifestyle. The isolation of mindfulness practice from other factors may reduce its effectiveness [111].

## 5. Mindfulness in Nature: How Nature Helps to Restore Resources in Order to Carry out Mindful-Based Practices

Environmental psychology rarely pays enough attention to the potential benefit that nature could bring to mindfulness training [113] or considers meditation practices as a way to facilitate and enhance restorative experiences [114]. Nevertheless, some authors, among which include Kaplan, one of the pioneers of the concept of restorative experiences [22,23], suggested some converging points between theory in the field of mindfulness and theory in restorative environments [115,116,117]. Both suggest disengaging from habitual and reactive thoughts and emotion (calling this mechanism detachment and being away, respectively) as a stress management strategy. Both suggest that attention underlies the positive effect of experiences. In particular, both theorize that present experiences (meditation and nature exposure) are characterized by a particular quality of attention (curiosity and soft fascination, respectively) [22,23,116,117]. Thus, experiences in nature can support meditative states through soft fascination (attentional state that restores resources effortlessly) and by being away. Meditation practices, in turn, can help people become positively engaged and curious toward restorative environmental conditions [113,115]. People could reduce stress through mindfulness meditation and exposure to nature by achieving psychological distance from stressors and distraction rather than addressing and eliminating them [118].

In addition to similarities, there are also some differences between the two approaches. One of the most evident is that mindfulness focuses on the voluntary practice (and effort) of specific individual skills to achieve the target (i.e., stress reduction). On the other hand, restorative theories claim that nature holds some characteristics that capture the involuntary attention of people. In other words, for mindfulness theories, the connection between present experience and stress management is a top-down attentional process driven by internal factors, while for restorative theories, it is a bottom-up attentional process driven by external factors. However, this difference is not a limitation for the tentative of integration between the two approaches. Lymeus et al. [118] proposed that these top-down and bottom-up processes can converge to enhance each other, supporting the mindfulness state and restorative processes. The approach to mindfulness training that draws on restorative qualities in natural environments could support beginners with stress or concentration problems in effortless meditation. When mindfulness practice requires attentional effort, for example, in beginner practitioners, exposure to natural environments could support effortless mindfulness-like states. Then, directing the practice toward nature scenes could offset that effort [118]. Reciprocally, practicing intentional curiosity can enhance practitioners’ ability to connect with natural stimuli, making them more fascinating (and, consequently, more restorative). Moreover, mindful detachment could help alleviate stress and worries, allowing people to enjoy the restorative potential of the natural environment more. In sum, meditation training and exposure to nature could complement each other when combined and not be a mere addition of independent effects [115].

### Experimental Evidence

The field of nature-based mindfulness is at its beginning and is not yet defined. However, research including both mindfulness and nature is a growing field. Many modern Buddhist meditation retreats and mindfulness retreats are held in natural environments [119,120]. Forest bathing (complete immersion in a natural environment) and mindfulness are often joined to maximize therapeutic effects [121,122,123]. Barriers, challenges, and difficulties toward achieving a mindful state could be overcome thanks to nature’s exposure [124].

A recent systematic review synthesizes results [125]. It includes only studies that examined the effect of real outdoor nature (no virtual and indoor nature). Only seven articles compared nature-based mindfulness with similar interventions but without contact with nature [118,126,127,128,129,130,131,132,133,134]. In three articles, the authors ask participants to “pay attention to purpose, in the present moment” (informal mindfulness) [126,127,128]. Two articles used horticultural therapy [130,131]. Shi et al. [129] used a mindfulness technique in which the subject focuses on his/her breathing and sensations while walking. Lymeus [118] used restoration skills training (ReST): weekly classes in a garden environment, with exercise instructions aimed to stimulate participants’ effortless, restorative transactions with the environment through sensory exploration, and practice in curiosity and detachment. Although the interventions’ characteristics varied widely concerning the type of mindfulness, in all studies, with the exception of one [131], mindfulness in the natural environment enhanced positive emotions or decreased negative ones. Its effect was superior to those of the control groups.

In all studies included in the previously reported review, mindfulness sessions in nature were only one element of the intervention (i.e., psychotherapy) to cope with stress [125]. However, studies considering only mindfulness intervention showed the same enhanced effect [132,133,134]. Formal (e.g., MBSR) and informal (e.g., walking mindfulness meditation) mindfulness training in the natural outdoor environment resulted in greater decreases in stress than in the other environments [132,134].

With respect to the mechanism of mindfulness, the literature suggested that stress is influenced primarily by mindful attention (actively sustaining attention to the present moment) and mindful acceptance (an attitude of openness, nonjudgment, and curiosity about the current moment) [133]. Moreover, people who receive a mindfulness intervention in nature reported higher decoupling ability and less negative emotions than those who walk indoors without mindfulness instruction. However, no difference was found from those who walk in nature without mindfulness instruction. This means that exposure to nature could also enhance the decoupling process typical of mindfulness practices [134]. Moreover, this result empirically supports the idea of a combined effect of mindfulness and nature [118]. Indeed, it suggests that only exposure to nature could not be sufficient for reducing negative emotions. Instead, by adding mindfulness intervention, negative emotions significantly decrease [134]. On the contrary, even if participants who also received mindfulness training reported greater awareness of their surroundings [134], mindful awareness (monitoring of present experiences, external and internal, both pleasant and unpleasant) did not directly influence perceived stress [133].

A largely separate line of research has recently provided indirect support for the notion that mindfulness can enhance nature contact and vice versa, suggesting that a mutually beneficial relationship between humans and nature can be mediated, in part, by mindfulness [135,136,137]. From one side, connection to nature can enhance mindfulness (in particular attention and acceptance) that, in turn, reduces stress [133]. Indeed, connection with nature increases only in mindfulness in the nature group and not in built mindfulness or indoor groups [132]. In particular, it could be that the tendency to attend mindfully to experiences in everyday life (i.e., with intentional curiosity) is related to a stronger general sense of connectedness with nature, whereas aspects of mindfulness more related to detachment may not be [138,139]. On the other side, using mindfulness to increase the awareness of restorative qualities of nature can activate a supplementary pathway to connectedness with nature [113] that, in turn, is positively associated with a better ability to cope with stress [140]. A recent review highlighted that connection with nature increases after exposure to nature (“forest bathing”), but it is significantly higher if participants were also engaged in mindfulness [122].

Considering WRS, no study has investigated the effect of mindfulness in nature intervention. One study [141] compares the effect of two interventions, the mindful emotion regulation (MER) and the savoring nature (SN) in strengthening the positive relationship between work engagement and proactive behaviors. The moderating role of supervisor justice was also considered. MER is the ability to remain aware despite the valence and magnitude of the emotions experienced. MER works with emotional regulation, allowing people to detach from their own emotions and observe them without judgmental attitudes. SN involves attending to nature voluntarily and effortlessly, the action that might have a restorative potential, according to the Attention Restoration Theory [23]. 

Molina and O’Shea’s SN intervention “involved reflecting on different natural images while listening to a piece of music” [141]. People in the MER intervention condition “received the same images and background music while listening to audio reflection activities” [141]. Results showed that both interventions promote prosocial behaviors, but only MER was effective when supervisor justice was low. The study highlights the important role of resources (supervisor justice) in determining the effectiveness of interventions at the organizational level. 

In conclusion, research on mindfulness has grown rapidly in the organizational context in recent years [85,111,142,143], as well as organizational attention to design healthy environment for workers [144,145,146,147]. However, further studies are needed to understand how (behavioral, cognitive, and affective mechanisms) situational factors (e.g., physical environment) may affect mindfulness practice and its beneficial effects on workers.

## 6. Discussion

In the previous paragraphs, we described how Mindfulness-Based Intervention (MBI) on one side and nature on the other side could help with coping with stressful situations at work. Moreover, we highlighted that nature-based mindfulness interventions are widespread but not in the workplace. To help researchers develop effective stress management intervention using mindfulness and nature, we propose a hypothetical model for coping with work-related stress by using Mindfulness in Nature-Based Intervention (MiNBI).

MBIs are designed to train individuals to promote mindfulness and to integrate its practice into daily life. Mindfulness, in turn, through the mechanisms that lie behind it and enhancing the described secondary processes, can affect resources and demands at work concerning the six main areas of work design: Demand, Control, Support, Relationship, Role, and Change. Mindfulness disrupts automatic thought processes and increases response flexibility, allowing for better problem solving and decision making involved in demanding and/or new situations. It promotes working memory, self-determination, and persistence, which are relevant for dealing with diverse demands. When facing competing demands, mindfulness promotes attentional control and efficiency. Enhancing cognitive capacity is linked with higher creativity and better problem-solving skills, which are important for crafting how individuals work effectively. Moreover, by fostering adaptive capacity and the adoption of new perspectives, together with empathy, mindfulness can contribute to healthier exchanges between individuals and increase backup behavior. Higher empathy, awareness, and intent in information processing promote the recognition of needs in others. Present moment awareness, attentional control, and better working memory contribute to clear perceptions of one’s role. In addition, cognitive flexibility can contribute to the systemic perception of how one’s role impacts and is influenced by others. Furthermore, by decreasing the use of automatic mental processes, mindfulness promotes the unbinding of cognitive schemas and broader possibilities for thinking and acting without being lodged in past schemas. Mindful people better adapt to new environments by finding new response strategies more efficiently.

**Figure 1:**
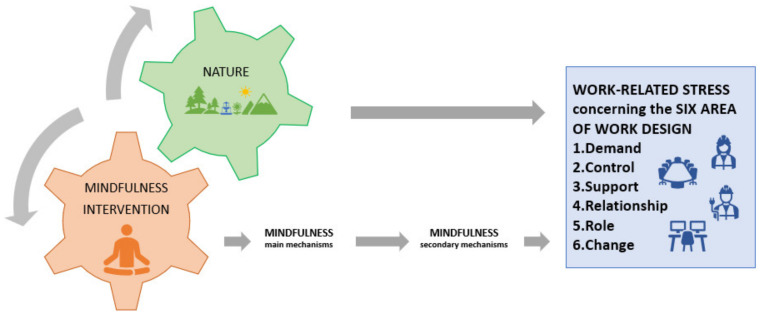
How does Mindfulness in Nature-Based Intervention works to mitigate work-related stress.

Nature acts in two different ways: On the one hand, nature directly affects stress and mental fatigue, which have a role in coping with demands and in increasing resources concerning the six areas of work design. Exposure to nature can reduce the negative mood state and enhance positive emotions, which in turn help in coping with stressful situations connected with high demands and increase social awareness so that individuals can relate with others more assertively and positively solve conflicts. Moreover, it contributes to mental fatigue restoration, with positive impacts on attention, increasing resources for controlling the way they develop their work, and helping in coping with highly demanding situations. Furthermore, exposure to nature has a calming effect, helping to reduce psycho-physiological activation during stress responses and protecting individuals against the impact of environmental stressors provoked by changes. On the other hand, nature can boost the effect of MBI in fostering mindfulness and, consequently, can improve all the positive effects of mindfulness that are already described. For example, contact with nature fosters cognitive performance, boosting the mindfulness’s effect on working memory, self-determination, and persistence, which are relevant for dealing with diverse demands and for the control of the way they develop their work. Moreover, contact with natural environments or elements as a part of mindfulness practice could boost the mindfulness effect on empathy, awareness, cognitive flexibility, and self-management skills of workers. The latter, in turn, contribute to developing positive interpersonal relationships, to the perception of their role within the organization, and to adjust effectively to change, reducing stress and promoting new response patterns. Figure 1 summarizes these mechanisms.

To develop these practices (MiNBI), workers could, for example, personalize personal workplace with nature elements and takes little breaks (e.g., 5 min every hour) to look at them (e.g., indoor plants, outdoor nature, or visual representations of nature). Alternatively, they can use break-time to have a short walk in a natural environment (e.g., outdoor nature, if available, or a terrace with plants, or towards a window with natural landscape). These actions could be occasions to use nature to restore their mental resources and also to practice mindfulness in natural exercises (e.g., pause for presence in nature, consciously relaxing in nature, focusing on nature, and open awareness in nature). 

MINDLIVEN (Mindfulness-in-Nature Based Training through Virtual Environments project) aims to develop a MiNBI that will include “formal” training with the following:

- Exercise: A dedicated time to practice a skill that takes about 10 min.
- Small exercise: A short exercise to practice a skill so it can be more easily fitted in the day, with a length of 2 or 3 min.
- Micro-practice: A very short exercise that lasts only about 15 s that can be very easily included in our day-to-day habits (e.g., before a challenging task as send an important e-mail or do a presentation).

Finally, management actions are critical for developing a healthy workplace and contributing to a context that facilitates individual actions to practice mindfulness and the use of nature. For this reason, we want to suggest some actions that managers should develop to deal with the demands and work characteristics that contribute to stress and, if not managed, will contribute to a decrease in performance and health problems. For example, we list the following:

1. Facilitate mindfulness practices in a specific place. Create a room for people to practice mindfulness with natural elements to recreate conditions that occur in nature, e.g., with an open view of natural landscape, water, natural sounds, essential oils, natural materials, and light that changes over time.
2. Facilitate mindfulness practices outdoors. Organize outdoor natural workspaces whenever possible.
3. Facilitate mindfulness practices without moving from the workstation. Place indoor natural elements where employees can easily see them from their workstations.

## 7. Conclusions

The workplace is one of the environments where people spend most of their day. Consequently, in addition to being a place of production, a workplace must also be a place that can generate wellbeing. In this paper, we summarized the positive effect of mindfulness and exposure to nature relative to workplace outcomes. Moreover, we highlighted the therapeutic potential of mindfulness in nature interventions also in the workplace. The proposed model will be used in future research studies as a theoretical framework to develop efficient interventions to cope with WRS. MiNBI could be a useful tool to enhance both production and wellbeing. However, it is important to underline that MiNBI should be part of a more comprehensive process for managing the risks of WRS that include also identifying work-related stress causes (i.e., risk evaluation).

## Data Availability

Not applicable.
